# Diving bradycardia of elderly Korean women divers in cold seawater: a field report

**DOI:** 10.1186/2046-7648-4-S1-A55

**Published:** 2015-09-14

**Authors:** Joo-Young Lee, Hyo-Hyon Lee, Siyeon Kim, Young-Joon Jang

**Affiliations:** 1 COM:FORT Laboratory, College of Human Ecology, Seoul National University, Seoul, Republic of Korea

## Introduction

Throughout the world, breath-hold diving has been reported in women divers in Korea (haenyeo) and Japan (ama), sponge divers in Greece, pearl divers in the South Pacific and shell divers in Australia. However, most of them have begun to use SCUBA (self-contained underwater breathing apparatus), whereas women divers in Korea and Japan have continued breath-hold diving. Since over 80% of haenyeos at present are in their sixties or older, it is predicted that breath-hold diving will cease to exist in a couple of decades. There is very little known about elderly haenyeos' cardiovascular changes. The purpose of the present study was to explore the diving patterns and heart rate of older Korean women divers while breath-hold diving in cold seawater.

## Methods

Nine haenyeos participated in a field study in Jeju Island, Korea [mean (SD) 68 (10) yr in age, ranged from 56 to 83 yr]. Air temperature, air humidity, and globe temperature were 14.9 (1.0) °C, 63 (4) % RH, and 18.3 (1.5) °C, respectively. Air flow on the coast was 3.4 (1.0) m.s^-1^. Seawater temperature was 10 to 13°C. We recorded heart rate at 1 min intervals as soon as the haenyeos began walking to the coast and until they returned to their preparatory house after diving.

## Results

Average (SD) diving time was 253 (73) min. Total frequency of dives was 97 (28) times (56~134 times) and they dived 23 (8) times per hour. All haenyeos showed diving bradycardia with a decreased heart rate of 20 (8) % at the bottom time (101 (20) bpm) when compared to surface swimming time (125 (16) bpm) in the sea. Older haenyeos had shorter diving time, less diving frequencies, and lower heart rate at work (p < 0.05).

## Discussion

Diving bradycardia is a physiologically protective oxygen-conserving mechanism common in diving mammals and breath-hold divers. For elite young male breath-hold divers the mean (SD) decline of heart rate was 44 (10) % [1]. Hong et al. [2] reported an approximate 30% decrease in heart rate during breath-hold diving for young haenyeos, while we found a 20% decrease in heart rate for the elderly haenyeos. The reductions in diving time, diving frequency and heart rate imply that haenyeos voluntarily adjust their workload along with advancing age and diminishing cardiovascular functions. Also, the elderly haenyeos in this study had relatively longer surface time in the sea than young haenyeos in previous studies, which is regarded as a behavioural adaptation to compensate for attenuated cardiovascular capacity due to ageing.

## Conclusion

This study confirmed that elderly haenyeos had diving bradycardia during bottom time, with shorter diving time, less diving frequencies and lower heart rate with their advancing age.
